# Applications of Nuclear Magnetic Resonance Sensors to Cultural Heritage

**DOI:** 10.3390/s140406977

**Published:** 2014-04-21

**Authors:** Noemi Proietti, Donatella Capitani, Valeria Di Tullio

**Affiliations:** Laboratorio di Risonanza Magnetica “Annalaura Segre”, Istituto di Metodologie Chimiche, CNR Area della Ricerca di Roma 1, Via Salaria Km 29,300, 00015 Monterotondo (Rome), Italy; E-Mails: noemi.proietti@cnr.it (N.P.); valeria.ditullio@cnr.it (V.D.T.)

**Keywords:** unilateral NMR, ^13^C-CPMAS, paintings, porous stones, moisture detection, non-invasive analysis, non-destructive analysis, cultural heritage

## Abstract

In recent years nuclear magnetic resonance (NMR) sensors have been increasingly applied to investigate, characterize and monitor objects of cultural heritage interest. NMR is not confined to a few specific applications, but rather its use can be successfully extended to a wide number of different cultural heritage issues. A breakthrough has surely been the recent development of portable NMR sensors which can be applied *in situ* for non-destructive and non-invasive investigations. In this paper three studies illustrating the potential of NMR sensors in this field of research are reported.

## 1. Basic of NMR Sensors

Nuclear magnetic resonance (NMR) is a powerful tool in many fields and a diversity of NMR measurements and methodologies have been and are currently being exploited. High resolution NMR spectroscopy in solution provides a method for determining the structure of molecules and macromolecules [1,2], whereas solid state NMR spectroscopy [3] is used for characterizing organic [4], inorganic [5], and hybrid materials [6]. Although magnetic resonance imaging (MRI) is a non-destructive diagnosis tool traditionally applied in clinical medicine, the application to materials [7] and food science [8] is now well established. High resolution magic angle spinning (HRMAS) NMR allows the investigation of “soft matter” [9]. Molecular motions can be studied by measuring relaxation times, and pulsed-gradients of magnetic field (PFG-NMR) are applied to investigate molecular translational diffusion [10].

Almost any element of the periodic table may be analyzed in the liquid, soft or solid state, the only limitation being natural isotopic abundance and sensitivity to the NMR experiment. To overcome the problem of sensitivity many NMR methodologies and sensors have been developed. For example, the reverse detection technique [11] increases the sensitivity in the detection of low abundant heteronuclei by an indirect way and makes possible the study of samples in low concentrations. Cryoprobes and microprobes [12,13] offer the chance to minimize the amount of material needed to perform the NMR analysis of soluble samples down to the microgram scale. High power decoupling, magic angle spinning and cross-polarization to enhance the sensitivity of rare nuclei have made it possible to investigate samples in the solid state [14]. The amount of material needed for solid state analysis has progressively decreased from 400–500 mg to a few mg. Promising NMR sensors and techniques in terms of their increased intrinsic sensitivity are under development such as micro-coils for MAS NMR applications [15], planar microslot waveguide NMR probes [16], para-hydrogen induced polarization (PHIP) [17], and dynamic nuclear polarization (DNP) [18].

The simplest NMR experiment consists of applying a radio-frequency (rf) pulse with a duration of a few microseconds to the sample. As the rf pulse is switched off, nuclei return back to equilibrium generating an interferogram known as free induction decay (FID). Provided that the magnetic field is homogenous and a Fourier transformation is applied to the FID a spectrum is obtained with peaks of appropriate width and frequency (chemical shift). In the frame of pulsed low resolution NMR, FID obtained after applying two or more pulses is used in the determination of relaxation times [19]. After perturbing a spin system with a proper rf pulse sequence, the system will return back to equilibrium through a process called “relaxation” characterized by a decay time constant known as relaxation time. The longitudinal relaxation time T_1_ is the decay time constant for the recovery of the z component M_z_ of the nuclear spin magnetization towards its thermal equilibrium value. Longitudinal relaxation is due to energy exchange between nuclear spins and the surrounding lattice re-establishing thermal equilibrium. The transverse relaxation time T_2_ is the decay time constant for the component M_xy_ of the nuclear spin magnetization in the xy plane. As spins move together, their magnetic fields interact slightly modifying their precession rate causing a cumulative loss in phase which results in transverse magnetization decay. Note that relaxation times depend on the physico-chemical properties of materials.

There is growing understanding that monitoring and diagnosis of artifacts are mandatory to prevent or at least delay their degradation. Because the amount of samples obtained from precious artifacts to be analyzed must be reduced to a minimum, multi-analytical approaches are advisable where micro-destructive, non-destructive, and possibly non-invasive techniques are combined. A breakthrough for application of NMR to cultural heritage has been the development of unilateral NMR sensors [20–23] which allow one to study arbitrarily sized objects non-invasively, combining open magnets and surface rf coils to generate a magnetic field external to the sensor and inside the object under investigation. Although the magnetic field of these sensors is inhomogeneous, it is possible to measure NMR parameters such as proton density, relaxation times, self-diffusion coefficient, and even to collect correlation maps [24] of unmovable and precious artifacts and monuments belonging to the cultural heritage. Because of the inhomogeneous field [25], the signal (FID) decays very quickly and cannot be directly detected. Therefore the signal must be recovered as an echo. Moreover, the inhomogeneous field is a further source of relaxation which makes the transverse relaxation time shorter than that measured in a homogeneous field.

**Figure 1.**
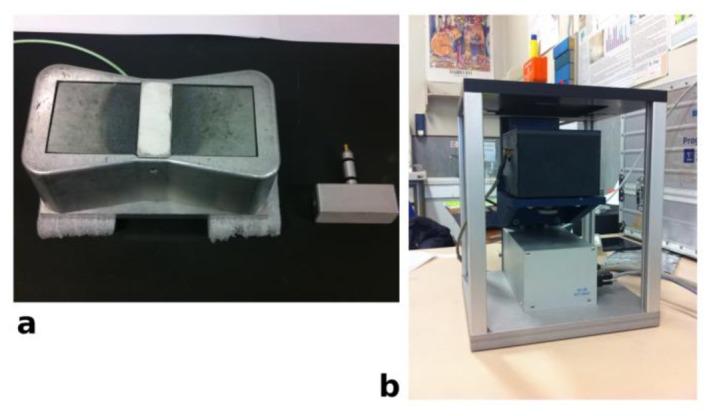
(**a**) Unilateral NMR sensor by Bruker Biospin; (**b**) NMR sensor with a uniform gradient to resolve the near surface structure of arbitrarily large samples, the sensor is placed on a lift that allows one to move the magnetic field inside the object to be analyzed with micrometric steps, sensor by RWTH Aachen University, Aachen, Germany [26].

Figure 1a shows a palm size NMR sensor. It consists of a U-shaped magnet obtained using two anti-parallel permanent magnets mounted on an iron yoke with the rf coil positioned in the gap of the magnet. The magnetic field is external to the device, enabling large objects to be studied without any sampling. Different probe heads, each tuned to the proper frequency are used to obtain different depths of measurement. A further development of unilateral NMR devices are sensors that can scan depths up to 2.5 cm, producing depth profiles with micrometric spatial resolution [26]. These devices generate an inhomogeneous magnetic field with a uniform gradient to resolve the near surface structure of arbitrarily large samples. To improve gradient uniformity, the device works at a fixed depth from the sensor, where high depth resolution can be achieved. The position of the excited slice inside the sample can be varied by displacing the sensor using a high-precision lift that repositions the magnet with respect to the sample. Figure 1b shows a device consisting of a permanent magnet mounted on a precision lift. Application of these devices has opened a number of new possibilities also in the field of cultural heritage [27–34].

In the following we report three cases illustrating the potential of NMR sensors in cultural heritage.

## 2. Quantitative Moisture Distribution Mapping in an Ancient Wall Painting

Water is a major cause of decay to building masonry in cultural heritage sites [35]. Determining the course and distribution of water through the wall is a fundamental step in conservation work and is particularly true for wall paintings. In fact wall paintings are constituted of materials having an open porosity resulting in an easy accessibility of liquids and gases such as salt solutions, atmospheric pollutants, dampness, and solutions of material used for conservation treatments. Wall paintings are part of an essentially open physical system due to contact with contiguous structures (walls, ground, roofs) that are dynamically involved in a series of physical and chemical events. A number of factors must be taken into account, such as the vulnerability of thin painted surfaces forming the interface between the plaster and the environment, the proximity of places crowded with people to the wall painting, the difficulty of controlling potential deteriogens, such as moisture, biological colonization, and pollution. Additionally, in many cases the surrounding microclimate cannot be controlled. The knowledge of water path and distribution through the wall is mandatory for determining the mechanism by which water triggers and accelerates damage, and for developing and planning interventions for conservation. Nevertheless, the amount and the distribution of moisture within a wall painting is difficult to determine. The methods currently used for this determination are IR thermography (IRT), electrical conductivity, and gravimetric tests. However IRT does not allow a quantitative evaluation of the moisture content, electrical conductivity may be affected by the presence of salts and gravimetric tests require the drilling of solid cores, which is strictly forbidden in the case of precious artworks such as wall paintings.

**Figure 2.**
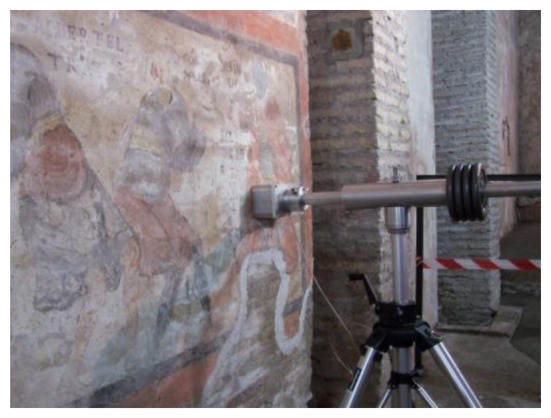
Portable unilateral NMR instrument measuring the moisture content in the wall painting *Saint Clement at Mass and the Legend of Sisinnius* in St. Clement's Basilica, Rome.

The availability of unilateral NMR sensors has allowed non-destructive and non-invasive mapping of moisture distribution in ancient wall paintings [36–39]. Figure 2 shows a portable NMR instrument measuring moisture content in the wall painting *Saint Clement at Mass and the Legend of Sisinnius* located in St. Clement's Basilica, Rome.

The wall painting, which dates back to 1080, is located on the second hypogeous level of the Basilica at a depth of about 6 m below the road level. The presence of a watercourse flowing under the foundation of the Basilica is one of the causes of the rising damp through the walls of the archeological site. Furthermore, microclimate conditions in the hypogeum are critical, with a very high relative humidity and low temperature during the whole year. The masonry and wall painting showed the characteristic degradation processes induced by a high level of humidity such as efflorescences, encrustations, and biological colonization. The damage due to action of water in the wall was so severe that reduction of dampness and stabilization of moisture content were mandatory before planning any restoration of the painted surface. To reduce rising damp in the masonry, a horizontal cut was carried out through the bricks of the wall just above floor level, and a hydrophobic mixture of polyester resin and marble powder was injected into the wall. The amount, distribution, and evolution of moisture in the wall painting were monitored by unilateral NMR before and after the intervention.

Measurements were carried out choosing a matrix of points on the painted surface. The same matrix of points was investigated before (February 2008) and after (February 2010) the intervention [36]. NMR measurements were carried out both on the superficial layers at a depth of 0.1 cm of the wall painting and at a depth of 0.5 cm in the plaster. Collected data were processed to obtain contour plots which are 2D representations of 3D surfaces. In these contour plots x and y are the coordinates of the measured region of the painted wall and z is the integral of the NMR signal, which is proportional to water content.

**Figure 3.**
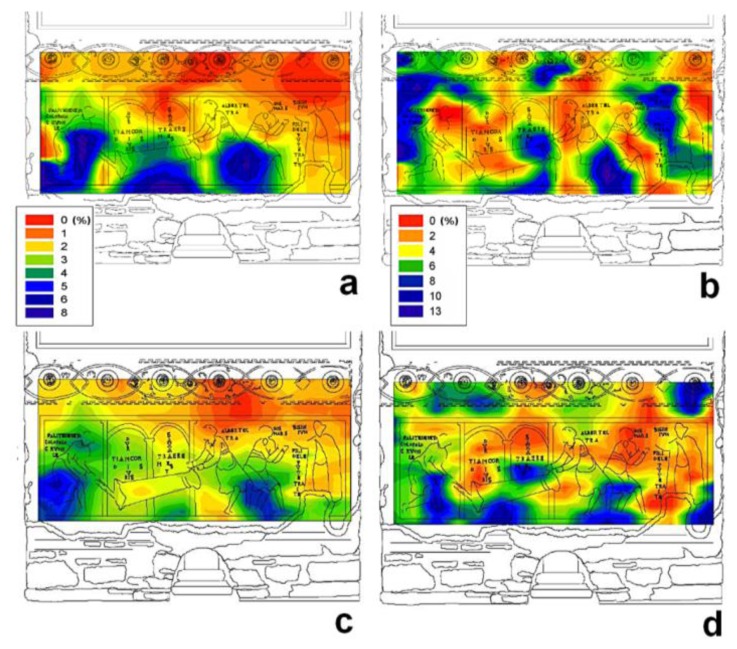
Moisture distribution maps obtained by unilateral NMR before (**a**,**b**) and after (**c**,**d**) intervention to reduce capillary rise of water through the wall. Left, maps collected at a depth of 0.5 cm, right, maps collected on superficial layer (0.1 cm) of the wall painting. Data adapted from [36].

Figure 3 shows moisture distribution maps obtained before (a,b) and after (c,d) the intervention to reduce the capillary rise of water. Maps collected at a depth of 0.5 cm are reported on the left whereas maps collected on the superficial layer of the wall panting are reported on the right. In these maps, the difference in the moisture level is encoded by a gradient of color, dark red indicates regions with the lowest moisture content, whereas dark blue indicates regions with the highest moisture content. Although the maps differentiated wall painting regions as a function of moisture content, a further step was necessary to calibrate the NMR signal and to assess the precise amount of moisture in each region. Briefly, the NMR signal was calibrated using four specimens prepared by restorers according to the ancient original recipe to reproduce the mortar used in the wall painting. This procedure allowed to scale the amount of moisture in each measured region. Furthermore, to validate the calibration procedure, NMR measurements and gravimetric tests were carried out on an unpainted area very close to the wall painting. Results obtained with these two techniques were found to be in very good agreement [36].

Maps obtained at a depth of 0.5 cm gave a clear image of the path of rising damp. The maximum water content measured in February 2010 (Figure 3c) seven months after intervention, was about 3% lower than that measured in February 2008 (Figure 3a) before intervention. Additionally, maps obtained after intervention clearly showed a lower rise of moisture and a net reduction in wet regions.

Maps obtained at a depth of 0.1 cm showed that maximum water content was 12%–13% in February 2008 (Figure 3b) and 11%–12% in February 2010 (Figure 3d). It is worth noting that these maps did not give information about the path of rising damp. In fact measurements at a depth of 0.1 cm regarded a slice very close to the painting-environment interface, which was mostly affected by the microclimate of the underground environment. The influence of microclimate on the quantitative moisture distribution in superficial layer of the wall painting was evident in the map collected November 2009 (data not reported), when the presence of a scaffolding caused a net lowering of relative humidity in proximity of the wall painting. In fact, monitoring of thermohygrometric parameters conducted simultaneously with NMR measurements showed that outside the scaffolding, mean relative humidity was about 98%, whereas inside it was about 88%, and the temperature inside the scaffolding was, on average, one degree higher than outside. In fact maps collected at a depth of 0.1 cm before (Figure 3b) and after intervention (Figure 3d) were found to be very similar to each other and little affected by the intervention. To summarize, the moisture content in the superficial layer of the wall painting was found to be largely affected by the microclimate of the hypogeous environment, whereas the moisture content at a depth of 0.5 cm was largely affected by the rising dampness through the wall.

### Experimental Details

Measurements were performed *in situ* with an unilateral NMR instrument from Bruker Biospin, which is a variant of NMR-MOUSE, see Figure 1a. NMR signal is the integral of the signal obtained applying a Hahn echo pulse sequence. Measurements were carried out at a depth of 0.1 cm from the surface of the wall painting and at a depth of 0.5 cm. In the former case, a probe head operating at 18 MHz with a π/2 pulse of 3 μs was used, whereas in the latter case a probe head operating at 16 MHz with a π/2 pulse of 10.4 μs, was used. In both cases the dead time was 15 μs. Measurements were carried on a matrix of 50 points, each point covering an area of 2 × 5 cm^2^. Collected data were processed to obtain a contour plot where x and y were the coordinates of the measured area of the wall painting, and z was the integral of the NMR signal. The detailed procedure for calibrating moisture distribution maps has been reported elsewhere [38,39].

## 3. NMR Stratigraphy of a Painting on Wooden Panel

Paintings consist of many layers such as pigments, binders, primer, varnishes, and so on. Knowledge of layers' structure or stratigraphy of the artwork provides information about the materials used, and the working practices of the artist. Because organic and inorganic components of the paint layer undergo degradation, the knowledge of composition is mandatory to assess suitable conservation and display conditions, to prevent or slow the decay process, and to plan restoration.

The common practice of obtaining information about stratigraphy is to cut cross-sections from the painting and analyze them by optical microscopy (OM) and scanning electron microscopy coupled with energy dispersive X-ray spectroscopy (SEM-EDS). Analytical techniques capable of identifying materials in paintings cross-sections such as chemiluminescent immunochemical imaging [40], surface-enhanced Raman spectroscopy [41], FTIR mapping [42], and secondary-ion mass spectrometry [43], have been developed.

NMR stratigraphy is an analytical technique which may be applied *in situ* to reveal different layers of a painting in a fully non-invasive manner. The first stratigraphy was published by Presciutti *et al.* [27]. With this technique, layers of different materials can be detected and their thickness can be measured. Because NMR stratigraphy does not require any sampling, many regions of the painting may be analyzed and monitored over time.

The stratigraphy encodes the amplitude of the ^1^H-NMR signal as a function of the depth scanned. The intensity of the signal indicating hydrogen content enables one layer to be differentiated from another. The sensor is placed on a lift that allows one to move the magnetic field inside the painting with micrometric steps.

**Figure 4.**
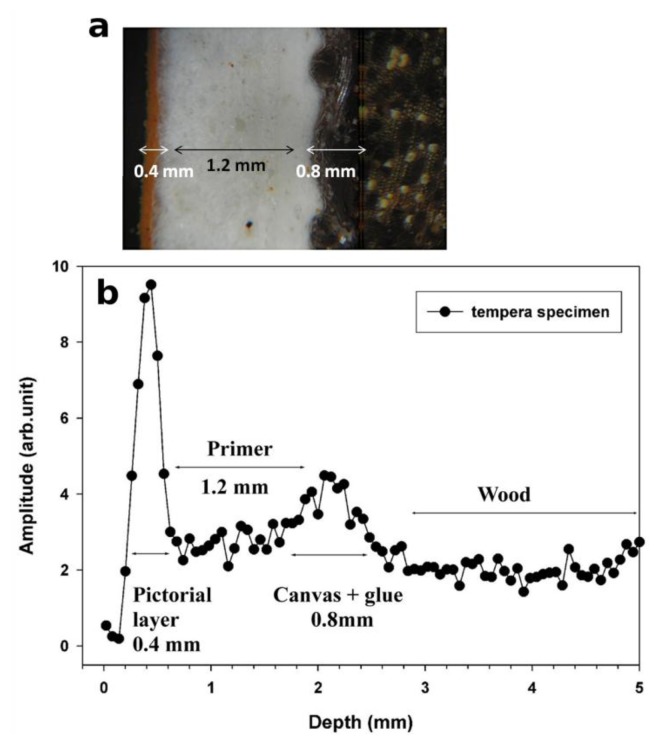
(**a**) Image across the painting layers obtained with an optical microscope on a cross section removed from the specimen; (**b**) NMR stratigraphy of a purposely prepared tempera specimen, the resolution of the stratigraphy is 50 μm.

To illustrate the performance of the NMR sensor, the stratigraphy obtained on a purposely prepared tempera specimen and the corresponding optical image are reported in Figure 4. Based on variation in signal intensity, the stratigraphy indicates four well-defined layers: the first (0.4 mm thick) is the pictorial layer, the second (1.2 mm thick) is the primer, the third is the *incamottatura* (canvas + glue, 0.8 mm thick), and the fourth one is the wood of the panel. The number of layers and their thickness match those observed by OM.

Based on relaxation times measured on the distinct layers detected by the stratigraphy, in principle it may be possible to qualitatively differentiate layers consisting of different types of organic material.

**Figure 5.**
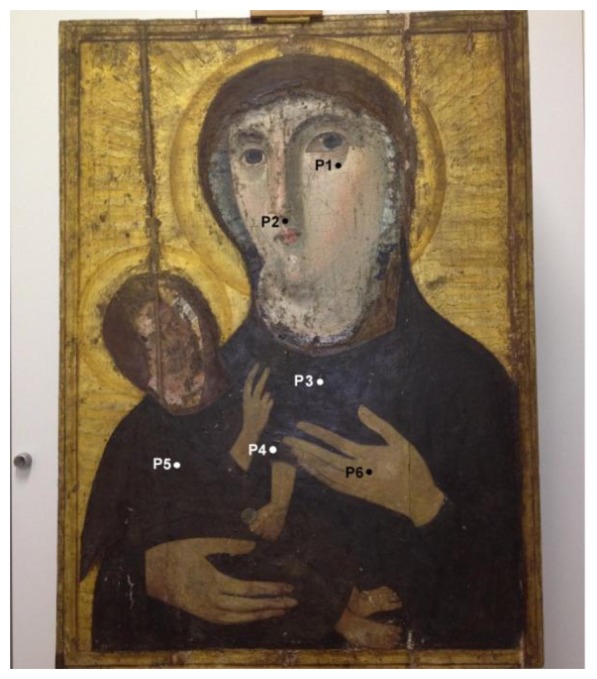
Icon and a few selected regions among those measured by unilateral NMR.

In the following we report a case study regarding an ancient icon that is thought to be a copy of the *Madonna Hodigitria* from Constantinople. It would have been transported from Troy to Rome in 1100 by Angelo Frangipane coming back from the Holy Land. As early as the 9th century it had probably been preserved in Santa Maria Nova church (nowadays Santa Francesca Romana church, Rome, Italy). The old painting was rediscovered in 1950 during a restoration work. Under a more recent painting dating from the 19th century, restorers discovered another painting dating back to the 13th century. Under this painting, two faces painted in encaustic on linen canvas were found, possibly dating back to the 5th century. Figure 5 shows the icon along with some regions scanned by portable NMR.

**Figure 6.**
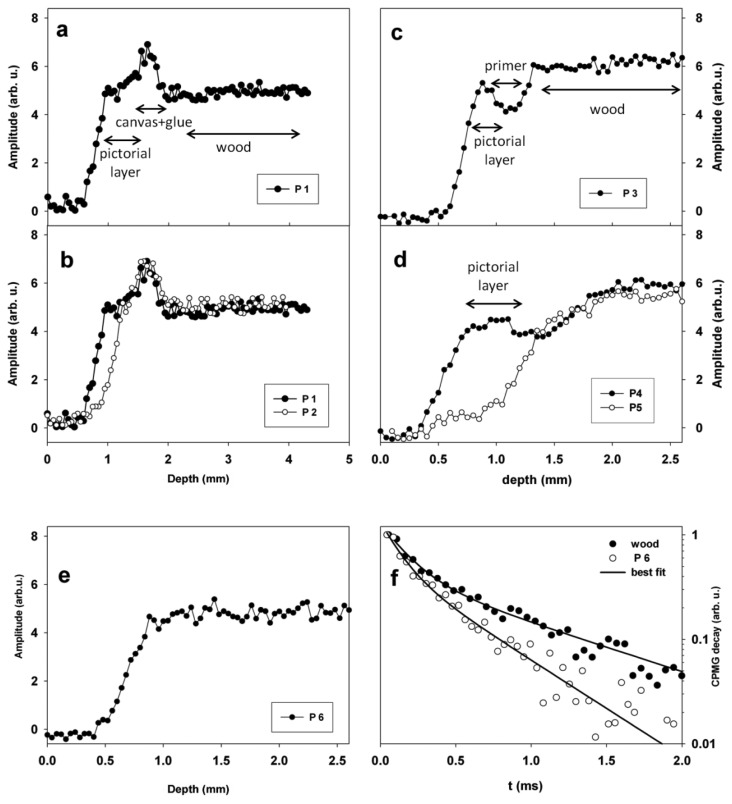
(**a–e**) NMR stratigraphies measured in different regions of the icon; (**f**) Comparison between CPMG decays measured on region P6 at a depth of 1 mm, and on the same region at a depth of 2.5 mm on wood.

Stratigraphies measured in these regions are reported in Figure 6. In Figure 6a a stratigraphy collected on the Virgin's face (region P1), is shown. The stratigraphy indicated the presence of three well defined layers, the first (0.5 mm thick) one was the pictorial layer, the second one (0.3 mm thick) was due to the presence of *incamottatura* (canvas + glue), and the third one was the wood of the panel. CPMG decays measured on Virgin's face were able to differentiate the three layers detected by stratigraphy. In fact two T_2_ values of 0.10 and 0.24 ms in a relative amount of 75% and 25% were found on the pictorial layer, whereas values of 0.15 and 0.40 ms in a relative amount of 84% and 16% were measured on the *incamottatura*. Eventually, two T_2_ values of 0.16 and 0.90 ms in a relative amount of 67% and 33% were measured on the wooden panel. These results indicated that layers detected by stratigraphy were made of different types of organic material.

In Figure 6b stratigraphies measured in regions P1 and P2 on Virgin's face are compared. The comparison clearly showed that in P2 the pictorial layer was reduced to about one half than that measured in P1. In fact region P1 was well preserved, whereas in P2 a lacuna was observed.

Figure 6c shows the stratigraphy measured on Virgin's mantle (region P3). Three layers were observed, the first one (0.4 mm thick) was the pictorial layer, the second one (0.5 mm thick) was the primer, and the third one was the wood of the panel. Note that the pictorial layer was found to be thicker (0.8 mm) on Child's mantle (P4) than on Virgin's mantle (P3), see also Figure 6d.

The stratigraphy obtained on region P5 on the Child's mantle showed a lacuna, clearly evidencing the absence of the pictorial layer, see Figure 6d. The stratigraphy obtained on the Virgin's hand (P6) was found to be completely different from those collected on other regions of the painting, see Figure 6e. In fact the stratigraphy did not show the presence of any distinct layers. It is worth to note that the CPMG decay measured in region P6 on the Virgin's hand at a depth of 1 mm was different from that measured on the wood in the same region at a depth of 2.5 mm, see Figure 6f. Specifically, two T_2_ values were obtained in both cases, however T_2_ values of wood were found to be 0.16 and 0.90 ms in a relative amount of 67 and 33% respectively, whereas at a depth of 1 mm values of 0.12 and 0.47 ms in a relative amount of 60 and 40% were found. Actually the longest T_2_ component differentiated between wood and the more superficial layer indicating the presence of two distinct materials.

Few fragments of the wooden panel were investigated by ^13^C-CPMAS NMR spectroscopy. This technique permits to obtain spectra of solid samples, and require sampling of about 8–10 mg of solid material provided that a sample holder with an internal volume reduced to 12 μL is used. ^13^C-CPMAS NMR spectroscopy is a very powerful and unique tool to investigate structural changes occurring in ancient wood. Wood is a complex natural composite material made of cellulose, hemicellulose, lignin and water. In hardwood lignin is mostly made of G (guaiacyl) units (aromatic units with one methoxyl group) and S (syringyl) units (aromatic units with two methoxyl groups), whereas in softwood lignin is mostly made of G units. S units are further labelled as S(ne) in non-etherified arylglycerol β-aryl ethers, and S(e) in etherified arylglycerol β-aryl ethers.

**Figure 7.**
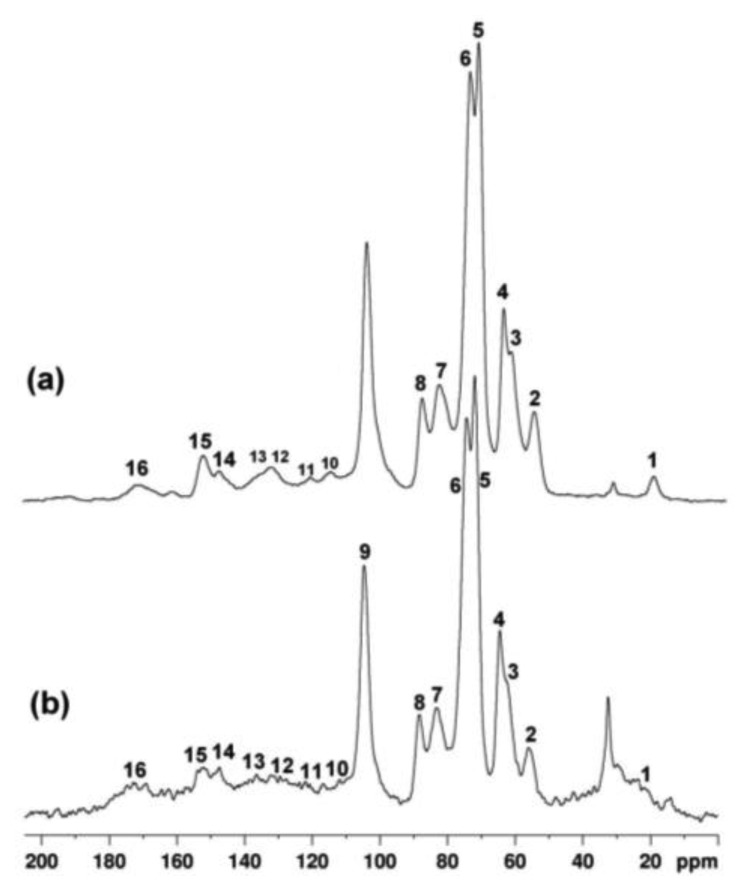
^13^C-CPMAS NMR spectra of a modern seasoned hardwood (**a**), and a fragment of wood sampled from the icon (**b**).

The integral and the frequency of each carbon resonance give information about the type of wood (hardwood or softwood), as well as indicating the state of degradation of the wood. In Figure 7 ^13^C- CPMAS NMR spectra of a modern seasoned hardwood (a), and a wood fragment obtained from the ancient icon (b) are compared. According to the literature [44], the weak signal at 21 ppm (1) is assigned to CH_3_ carbon of the acetyl group in hemicelluloses. The peak at 32 ppm is tentatively assigned to polymethylene chains, e.g., cutin or waxes associated with the cuticle [45,46]. The peak at 55.6 ppm (2) is assigned to methoxyl groups of aromatic units of lignin. The region between 60 and 105 ppm is dominated by the intense peaks mostly assigned to cellulose (3–9). The region between 105 and 160 ppm is specific to the aromatic carbons of lignin (10–15). The signal at 172 ppm is assigned to acid groups possibly present in wood and to carbonyls of acetoxy groups of hemicelluloses. The resonance at 152.6 ppm (15) is assigned to carbon atoms C3 and C5 of S(e) units, namely S3(e) and S5(e), and resonance at 147 ppm (14) is assigned to carbon atoms C1 and C4 of G units, namely G1 and G4, and to carbon atoms C3 and C5 of S(ne) units, namely S3(ne) and S5(ne). In degraded wood, the ratio of the integral of resonances 15 and 14 allows one to estimate the depletion of β-O-4 linkages in lignin. The integral of each resonance was obtained by applying a deconvolution procedure to spectra.

In our case the ratio I(15)/I(14) was found to be similar in seasoned wood and in the fragment of the icon, being 1.4 and 1.5 respectively. The relative amount of carbohydrates and lignin can be evaluated taking the integral of resonance 9 at 104.8 ppm I(9) (anomeric carbon of cellulose) as reference and the integral of resonance 2 at 55.6 ppm, I(2) (methoxyl groups of lignin). In seasoned hardwood the average value of the ratio between I(9) and I(2) was found to be about 2, whereas in the wood of the icon this ratio increased to 2.95 indicating a loss of lignin component with respect to cellulose, possibly due to the action of microrganism preferentially acting on lignin. Finally, in sample obtained from the icon peaks of lignin resonating between 120 and 180 ppm were found to be broadened possibly indicating the occurrence of chemical rearrangements inside the biopolymers network.

To summarize, this study illustrated how NMR stratigraphy is a powerful tool to analyze paintings characterized by the presence of regions transformed using different techniques in different periods. The comparison among the collected stratigraphies allowed the detection of three painting techniques, the first concerning the oldest period dating back to 5th century (Virgin and Child's faces), the second corresponding to the 13th century and the third one probably related to a restoration carried out to fill in missing regions. Stratigraphies collected at deeper depths on Virgin and Child's mantles painted in the 13th century did not reveal the presence of any previous painting allowing one to confirm the absence of over-painted regions. The absence of over-painted regions and the presence of different artistic techniques confirmed that in the past only the Virgin and Child faces painted in encaustic on linen canvas were cut from the original painting, saved and glued on a new wooden support. Values of transverse relaxation times obtained on different layers detected by the stratigraphy allowed us to qualitatively differentiate the organic materials that had been used in various periods. Information obtained by this analysis may also assist art historians in interpreting the artwork and more specific dating.

Results obtained by ^13^C-CPMAS NMR spectroscopy allowed the identification of the wood of the icon as a hardwood, indicated a loss of lignin component in the investigated region, and the occurrence of chemical rearrangements in lignin structure caused by degradation.

### Experimental Details

NMR stratigraphy and relaxation times were collected at 13.62 MHz with a portable NMR instrument from Bruker Biospin interfaced with an unilateral sensor by RWTH Aachen University, Aachen, Germany, see Figure 1b [26]. Experiments were carried out by repositioning the sensor in steps of 50 μm to cover the desired spatial range, from the outermost surface of the painting to a depth of 0.25 cm with a resolution of 92 μm or to a depth of 0.45 cm with a resolution of 57 μm. In the former case, the π/2 pulse was 6.8 μs and 512 scans were collected. In the latter case the π/2 pulse was 11 μs and 1,024 scans were collected. To obtain stratigraphy exclusively dependent on the proton spin density the intensity of each experimental point was obtained as the average of the intensity of the first four echoes acquired with a CPMG sequence. Transverse relaxation times were measured at selected depths with the CPMG sequence, 128 echoes were recorded with an echo spacing of 47 μs. The shortening of transverse relaxation time caused by the inhomogeneous field was minimized by using an echo spacing as short as possible [25]. Data obtained were normalized and then fit to the following equation:

(1) $$\text{Y}(\text{t})=\sum_{i=1}^{\text{n}}{\text{W}}_{\text{i}}{\text{e}}^{\frac{-\text{t}}{{\text{T}}_{2\text{i}}}}$$

where *n* is the number of components of the decay of magnetization, *W*_i_ is the weight of the *i*th component (spin population), and *T*_2i_ is the relaxation time of the *i*th component. Note that the sum of spin populations was normalized to 100%.

Solid-state ^13^C-CPMAS NMR spectra were recorded with a Bruker Avance III spectrometer operating at the proton frequency of 100.63 MHz. The spin rate was 12 kHz. A Sample of the wooden panel (about 8 mg), and a sample of modern seasoned hardwood were cut into small pieces and packed in 4-mm zirconia rotors with the available volume reduced to 12 μL. The contact time for the cross-polarization was 1.5 ms, recycle delay 3 s, ^1^H π/2 pulse 3.5 μs, 12,000 scans were collected. High-power proton dipolar decoupling was carried out using the Spinal-64 scheme [47]. The decoupling field was 140 kHz. Spectra were acquired with a time domain of 1,024 data points were zero filled and Fourier transformed with 2048 data points applying exponential multiplication with 8-Hz line broadening.

## 4. NMR Investigation of a Consolidating Treatment on a Porous Stone

Consolidation is carried out on stone materials affected by loss of cohesion with the aim of re-building them. This is normally achieved through the permeation of solubilized agents in stone material, close to the surface, as in most of cases, loss of cohesion is limited to regions located near the surface. Often consolidating treatments having also a hydrophobic action are chosen to prevent water penetration into the stone. The treatment should assure vapour leakage, avoid modifications in the optical properties of material, improve resistance, and delay future degradation processes. Furthermore, the compound used to consolidate the stone should not accumulate in specific regions of the stone to avoid the formation of inhomogeneous regions where impregnated layers and layers underneath might differently respond to changes in thermohygrometric condition or mechanical stress. The penetration depth of the treatment into the stone is also an important parameter for evaluating the treatment. Direct and indirect methods [48] may be used at this aim. Usually, direct methods measure the amount of applied product by instrumental chemical analysis, primarily with analytical techniques such as FTIR, SEM-EDS, and x-ray photoelectron spectroscopy (XPS);, these methods are often destructive and cannot be applied directly *in situ*. Indirect methods involve measuring the physical properties modified by the treatment. In the case of water repellent product such modifications are connected to variation in hydrophobic action and physical properties related to interaction with water. These methods include measurement of contact angle, water drop absorption time, acid etching, capillary water absorption, and water absorption through a pipe. In the case of product with consolidating properties, indirect methods usually include measuring physical-mechanical parameters, *i.e.*, bending strength, splitting tensile strength, modulus of elasticity, drilling resistance, and ultrasonic velocity.

With unilateral NMR sensors information is obtained provided that the consolidating treatment has also an hydrophobic action or the treatment appreciably fills pores up. In this case water (or other liquids) is used as contrast agent to obtain information about the treatment. Specifically, water-saturated untreated and treated stone specimens may be investigated by NMR to evaluate the amount of water present into the porous structure, and the water mobility and distribution into the porous structure [49–51]. From this investigation information may be obtained about the penetration depth of the treatment, hydrophobic action, occurrence of inhomogeneities in the treated stone, and changes in pores size distribution caused by the treatment.

**Figure 8.**
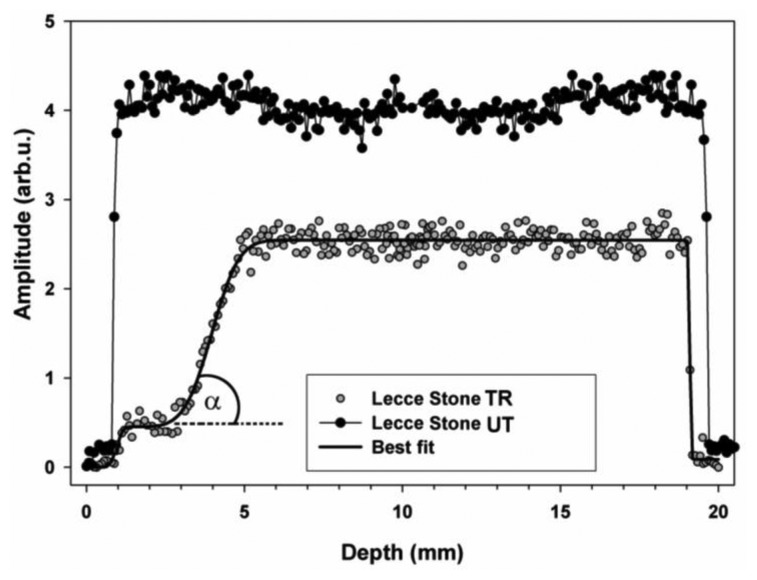
Depth profiles of untreated Lecce stone (UT), and Lecce stone treated with HDDA polymerized *in situ* (TR) obtained after making specimens absorb water by total immersion. The solid line through experimental points was obtained fitting the profile to Equation (3).

In the case reported here an NMR sensor with a uniform gradient was used to obtain ^1^H depth profiles before and after a consolidating treatment with monomer 1,6-hexanediole diacrylate (HDDA) polymerized *in situ*. ^1^H depth profile encodes the amplitude of the NMR signal as a function of the depth scanned. Figure 8 compares the depth profiles measured in untreated and treated Lecce stone obtained after making the specimens absorb water by total immersion.

To understand the meaning of depth profiles it must be born in mind that the amplitude is directly proportional to the amount of absorbed water. As a consequence, high amplitude corresponds to a high amount of absorbed water, whereas low amplitude corresponds to a low amount of absorbed water [31]. Comparing the amplitude of depth profiles regarding untreated and treated specimens it is possible to obtain information about the amount of water absorbed by specimens and, consequently, to scale the hydrophobic action of treatments and/or the reduction of pores volume accessible to water. Compared to the amplitude of the profile of the untreated specimen, the amplitude of the profile of the treated specimen was very reduced in the first 3 mm, indicating that water absorption was strongly impaired due both to pores coating that reduced pores volume available to water and to the hydrophobic action caused by the change of surface capillary absorption properties of the stone. At deeper depths the amplitude started increasing. From 5 to 19 mm the amplitude was rather constant and always lower than that measured in the untreated specimen, indicating that the amount of adsorbed water was reduced. Parameters obtained by fitting the profile to Equation (3) are reported in Table 1 along with the slope at the inflection point calculated from Equation (4).

Note that the fast rising initial part of the profile is very close to water–air–specimen interface. As a consequence, because affected by surface effects, parameters *x*_1_, Δ_1_, and b_1_ which encoded this part of the profile were no further considered. The penetration depth of the treatment was found to be *x*_2_ = 3.96 mm.

Slopes at inflection point *b*_k_ are used to encode the fastness of the amplitude variation of the profile. In fact, the greater the *b*_k_ value, the sharper the amplitude variation. Note that a very rapid amplitude variation indicates the occurrence of inhomogeneous regions due to the treatment. Therefore slopes at inflection points along with angle of inclination α_k_ give important information regarding the dispersion of the treatment in the specimen. Furthermore these parameters may also be compared with those which would be obtained in the presence of very sharp inhomogeneities caused by the accumulation of the compound used to treat the stone.

**Figure 9.**
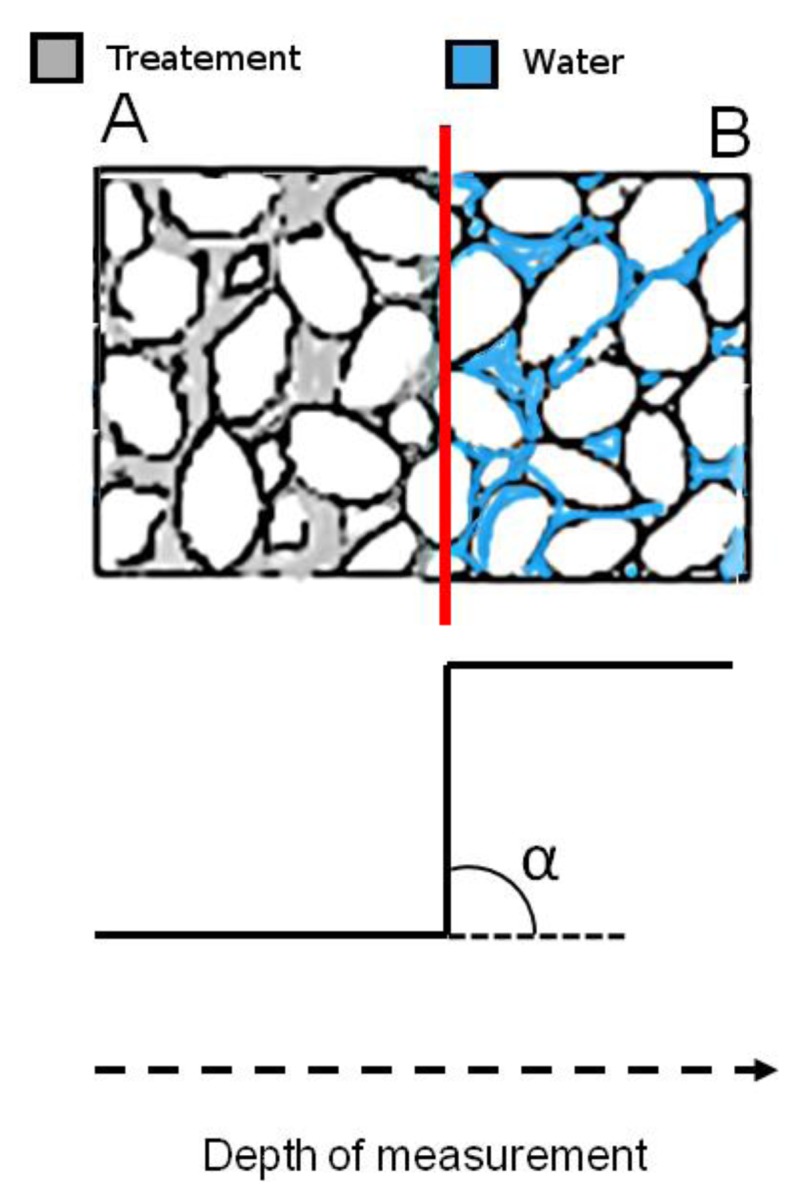
Top, sketch of an inhomogeneous region in a treated stone. Bottom, ^1^H depth profile encoding the inhomogeneous region.

For the sake of clarity, an inhomogeneous region which may form in a consolidated stone is sketched in Figure 9, top. In region A the water absorption is impaired by the treatment, whereas in region B the treatment is no longer effective to impair water absorption. The interface between A and B is a region of sharp discontinuity where treated layers come in contact with fully untreated layers. The occurrence of an inhomogeneous region like that sketched in Figure 9 top, corresponds to the ^1^H NMR depth profile reported in Figure 9 bottom. The intensity of the depth profile is very low in region A where water absorption is impaired, at the interface the intensity abruptly increases to reach the high value detected in region B where water absorption is no longer impaired. Note that the value of the slope approaches infinity and the angle of inclination is 90°.

The case sketched in Figure 9 is in principle one of the worst cases which may occur in a consolidated stone. Smoother transitions from treated to untreated layers indicating a better dispersion of the treatment in the porous matrix, are required, with slope b_k_ far from infinity, and angle of inclination α_k_ ≪ 90°. As a matter of fact *b*_k_ and α_k_ are parameters that may be used to compare treatments or for assessing that a treatment does or does not give rise to sharp inhomogeneities.

In treated Lecce stone the slope b_2_ was found to be 1.54 mm^−1^, which corresponds to an angle of inclination α of 57°, indicating the presence of a region about 3 mm thick where the product used to treat the stone accumulated blocking up pores. This region was followed by another region where the amount of adsorbed product definitely decreased, making possible the absorption of a considerable amount of water.

**Figure 10.**
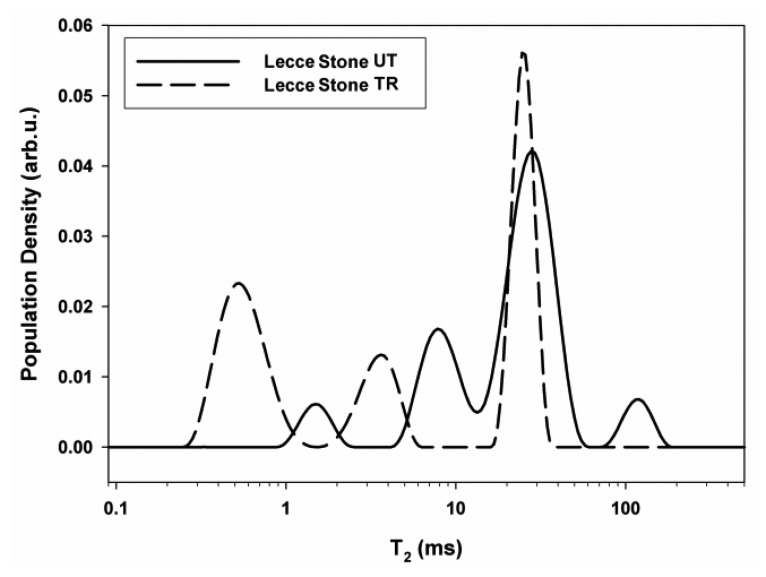
Transverse relaxation time distributions measured at a depth of 2 mm in Lecce stone untreated (UT) and treated (TR) with HDDA polymerized *in situ*.

Changes in pores size distribution caused by the treatment were evaluated through transverse relaxation time distributions. Figure 10 shows the comparison between the distribution obtained at a depth of 2 mm in untreated and treated Lecce stone.

The intensity of CPMG decay extrapolated at zero echo time was used to evaluate the amount of water adsorbed by the stone before and after the treatment:

(2) $$\Delta {\text{M}}_{0}=\frac{{\text{M}}_{0}^{\text{u}}-{\text{M}}_{0}^{\text{t}}}{{\text{M}}_{0}^{\text{u}}}\times 100$$

where ${\text{M}}_{0}^{\text{u}}$ and ${\text{M}}_{0}^{\text{t}}$ are intensities obtained for the untreated and treated stone respectively.

ΔM_0_ was found to be 66 % indicating that at a depth of 2 mm the treatment reduced the amount of absorbed water of about 66 %.

In the untreated specimen a peak centered at about 125 ms indicated the presence of water in large pores [52] in an amount of 8%. Two peaks centered at about 29 (64%) and 8.2 ms (23%) were due to water confined in medium pores, and the peak at about 1.5 ms (5%) indicated the presence of water in small pores. The major amount of water, 87%, was found to be confined in medium pores. After the treatment only three peaks were observed centered at 25, 3.77, and 0.55 ms with relative spin populations of 44%, 17%, and 39% respectively, whereas the peak centered at the longest value was lacking. Furthermore all peaks showed a shift to shorter values, and the amount of water in small pores increased from 5% to 44%.

### Experimental Details

Lecce stone specimens with a size of 5 × 5 × 2 cm^3^ were used. Specimens were treated by polymerizing *in situ* the pure monomer 1,6-hexanedioldiacrylate (HDDA). *In situ* polymerization [53] was carried out in acetone solution (20% *v*/v). The polymerizing system was absorbed from the specimen by capillarity. The specimen was placed on a thick layer of cotton soaked in the reaction solution for 4 h at 4 °C, in the absence of light. Polymerization was carried out for 24 h at 50 °C. After the polymerization, traces of solvent and unreacted monomers were removed by air evaporation.

Measurements were carried out at 13.62 MHz with an unilateral NMR instrument from Bruker Biospin interfaced with a single-sided sensor by RWTH Aachen University, Aachen, Germany [26]. Depth profiles of untreated and treated Lecce stone specimens were obtained with an echo spacing of 80 μs, and a nominal resolution of 23 μm. Profiles were acquired by repositioning the single-sided sensor in steps of 80 μm to cover the desired spatial range, from the surface of the specimen to a depth of 20 mm.

Transverse relaxation times T_2_ were measured using the CPMG pulse sequence, 4,096 echoes were recorded with an echo spacing of 43 μs. The error function was found to be suitable to fit the variation of water content in the porous matrix as a function of the depth scanned. This type of function has been successfully used to fit the intensity of ^1^H-NMR signal of a fluid confined into a porous structure vs temperature (IT-plot) [54–56] A similar equation has been previously used to fit the volume of intruded mercury V vs inverse pore dimension 1/R measured in cement paste by MIP [57].

Depth profiles obtained were fit to the following equation

(3) $$\text{f}(\text{x})=\sum_{\text{k}=1}^{\text{N}}\frac{{\text{w}}_{\text{k}}}{2}\text{erfc}\left|\frac{{\text{x}}_{\text{k}}-\text{x}}{\sqrt{2}\Delta_{\text{k}}}\right|+{\text{q}}_{0}$$

where N is the number of transitions of the amplitude in the depth profile, x_k_ and w_k_/2 are the abscissa and the ordinate of k component at the inflection point respectively, Δk is the half width of the transition of the amplitude from low to high value. The abscissa at the inflection point x_k_ is the penetration depth of the treatment. The slope at inflection point b_k_ that encodes the fastness of the variation of the amplitude of the profile, was calculated using parameters obtained from the best fit procedure:

(4) $${\text{b}}_{\text{k}}=\frac{{\text{w}}_{\text{k}}}{2\;\Delta_{\text{k}}}$$

The angle of inclination associated to b_k_ is: α_k_ = arctan |b_k_|. A regularized inverse Laplace transformation [58,59] was applied to echo envelopes obtained from the CPMG pulse sequence. With this representation the maxima of the peaks correspond to the most probable T_2_ values, and the peak areas correspond to the population of each component. Peaks centered at different T_2_ values correspond to water confined in pores of a different size.

## 5. Conclusions

In this paper the use of NMR sensors for characterizing and monitoring cultural heritage objects was illustrated. The three cases reported, though not exhaustive, demonstrate that NMR applications can be successfully extended to different issues regarding cultural heritage.

Unilateral NMR was used to quantitatively and non-invasively map moisture distribution and its evolution in an ancient deteriorated wall painting before and after intervention to reduce the capillary rise of water through the wall.

NMR stratigraphy enabled detection of different layers of an icon and measurement of the thickness of each layer, whereas transverse relaxation time measurements allowed one to reveal the presence of different types of organic material. This technique can be used to obtain information on the pictorial technique used by artist, state of conservation of the painting, and materials constituting the artifact.

^13^C-CPMAS NMR spectroscopy was used to investigate the state of conservation of the wooden panel of the ancient icon, revealing a loss of lignin and the occurrence of chemical rearrangement inside the lignin network.

An NMR sensor was used to investigate a consolidating treatment on a porous stone. The study was aimed at answering general questions such as the penetration depth of treatment into the porous material, its capacity to prevent water absorption, how treatment may change porosity of the stone, and how treatment may affect diffusion of water inside a porous structure. Moreover, in the case reported here, ^1^H depth profile indicated the presence of an inhomogeneous region where the product used to treat the stone accumulated blocking up pores. All obtained parameters provided important information regarding treatment performance.

Although NMR has not yet been widely applied in this field, it may also play a major role in the field of cultural heritage. The use of portable sensors for investigating large objects *in situ* without any sampling, combined with the use of laboratory sensors that require ever smaller amounts of sample foreshadow that NMR will probably become more and more competitive with other analytical techniques for the analysis of items belonging to cultural heritage.

## Figures and Tables

**Table 1.** Parameters obtained by fitting the profile of treated Lecce stone to Equations (3) and (4).

**Specimen**	***b*_1_ (mm^−1^)**	***b*_2_ (mm^−1^)**	***x*_1_ (mm)**	Δ**_1_ (mm)**	***w*_1_ (arb.u.)**	***x*_2_ (mm)**	Δ**_2_ (mm)**	***w*_2_ (arb.u.)**	***R*^2^**
Lecce stone treated with HDDA	1.53 ± 0.04	1.54 ± 0.03	0.95 ± 0.03	0.15 ± 0.07	0.45 ± 0.03	3.96 ± 0.03	0.68 ± 0.04	2.09 ± 0.03	0.989
